# Insights From Art Therapists on Using AI-Generated Art in Art Therapy: Mixed Methods Study

**DOI:** 10.2196/63038

**Published:** 2024-12-04

**Authors:** Fereshtehossadat Shojaei, Fatemehalsadat Shojaei, John Osorio Torres, Patrick C Shih

**Affiliations:** 1 Luddy School of Informatics, Computing, and Engineering, Indiana University Bloomington, 700 N Woodlawn Ave, Bloomington, IN, 47408, United States, 1 8128565754; 2 School of Computer Science, State University of New York, Oswego, NY, United States; 3 Center for Health Innovation and Implementation Science, School of Medicine, Indiana University, Indianapolis, IN, United States

**Keywords:** art therapy, artificial intelligence, AI, therapeutic interventions, assistive AI, engagement, health care, therapy, art, therapists' insights, daily life, practitioner, assistive, AI-generated image, accessibility, therapy sessions, AI-generated tool

## Abstract

**Background:**

With the increasing integration of artificial intelligence (AI) into various aspects of daily life, there is a growing interest among designers and practitioners in incorporating AI into their fields. In health care domains like art therapy, AI is also becoming a subject of exploration. However, the use of AI in art therapy is still undergoing investigation, with its benefits and challenges being actively explored.

**Objective:**

This study aims to investigate the integration of AI into art therapy practices to comprehend its potential impact on therapeutic processes and outcomes. Specifically, the focus is on understanding the perspectives of art therapists regarding the use of AI-assisted tools in their practice with clients, as demonstrated through the presentation of our prototype consisting of a deck of cards with words covering various categories alongside an AI-generated image.

**Methods:**

Using a co-design approach, 10 art therapists affiliated with the American Art Therapy Association participated in this study. They engaged in individual interviews where they discussed their professional perspectives on integrating AI into their therapeutic approaches and evaluating the prototype. Qualitative analysis was conducted to derive themes and insights from these sessions.

**Results:**

The study began in August 2023, with data collection involving 10 participants taking place in October 2023. Our qualitative findings provide a comprehensive evaluation of the impact of AI on facilitating therapeutic processes. The combination of a deck of cards and the use of an AI-generated tool demonstrated an enhancement in the quality and accessibility of therapy sessions. However, challenges such as credibility and privacy concerns were also identified.

**Conclusions:**

The integration of AI into art therapy presents promising avenues for innovation and progress within the field. By gaining insights into the perspectives and experiences of art therapists, this study contributes knowledge for both practical application and further research.

## Introduction

Creative arts therapies (CATs), encompassing modalities such as art therapy, dance or movement therapy, drama therapy, music therapy, psychodrama, and poetry or bibliotherapy, have demonstrated benefits for psychological and physiological health [1]. These therapies allow individuals to communicate their thoughts and feelings through artistic mediums serving as transitional objects [2]. Each CAT modality deploys distinct tools, using metaphor, creativity, imagery, symbolism, and nonverbal cues as therapeutic tools [3]. Specifically, art therapy uses visual art media within a psychotherapeutic context to facilitate both nonverbal and verbal self-expression and reflection through art-making, offering a structured and secure environment for playful experimentation and self-awareness, thereby promoting mental well-being [1,3].

The Fourth Industrial Revolution, characterized by advancements such as big data and artificial intelligence (AI), has spurred innovation in the medical field [4]. AI can analyze artwork, facilitate client engagement, and improve communication between therapists and clients [5]. For instance, AI-assisted systems provide insights into clients’ emotional states and therapeutic needs, therefore enhancing communication between therapists and their patients [6-8]. Machine learning capabilities allow AI to learn, sense, reason, and act autonomously, analyzing datasets and predicting attributes, thereby empowering practitioners and improving patient experiences [9].

Integrating AI into art therapy facilitates novel approaches for human-AI cocreation, enhancing creative expression [10]. Technologies such as DeepThInk, developed in collaboration with professional art therapists, promote user creativity while lowering barriers to artistic proficiency within human-AI cocreation [11]. Programs such as Coco Sketch, Drawing Apprentice, DuetDraw, GauGAN, and SmartPaint enable collaborative art-making, while mobile apps such as Mind Palette merge art therapy with generative AI to support mental health [11-15]. However, challenges persist, including uncertainty and output complexity persist in the integration of AI technologies into art therapy tools [11]. In addition, maintaining confidentiality is crucial, necessitating robust privacy measures in AI systems deployed in therapy contexts [16,17]. More generally, the shift to web-based therapy sessions, accelerated by the COVID-19 pandemic, has resulted in technology-related issues such as a steep learning curve [2,8].

While previous research has predominantly focused on leisure art-making [12,14,18,19], the meaningful incorporation of human-AI cocreation in art therapy remains relatively unexplored [11]. Insights from art therapists regarding AI-assisted tools are insufficiently researched [8], representing a significant opportunity for innovation in the field. This study aims to explore the use of AI in art therapy through co-design and interviews with art therapists who have integrated various technological tools into their practice. By examining their perspectives, challenges, and accomplishments, this research seeks to provide a comprehensive understanding of AI’s potential implications for the therapeutic process.

## Methods

### Overview

The methodology used in our study integrated design thinking and co-design approaches, which have been validated in previous scholarly work [20-22], followed by semistructured interviews. The initial steps of this first study included developing the user persona of an art therapist, creating the initial design prototype, and conducting the one-on-one co-design sessions. These sessions involved 10 art therapists, followed by semistructured interviews to gather their feedback on the design, which will be the focus of the second phase of this study in the future (see Multimedia Appendix 1 for co-design protocol and Multimedia Appendix 2 for semistructured interview questions).

### User Persona

**Figure 1.**
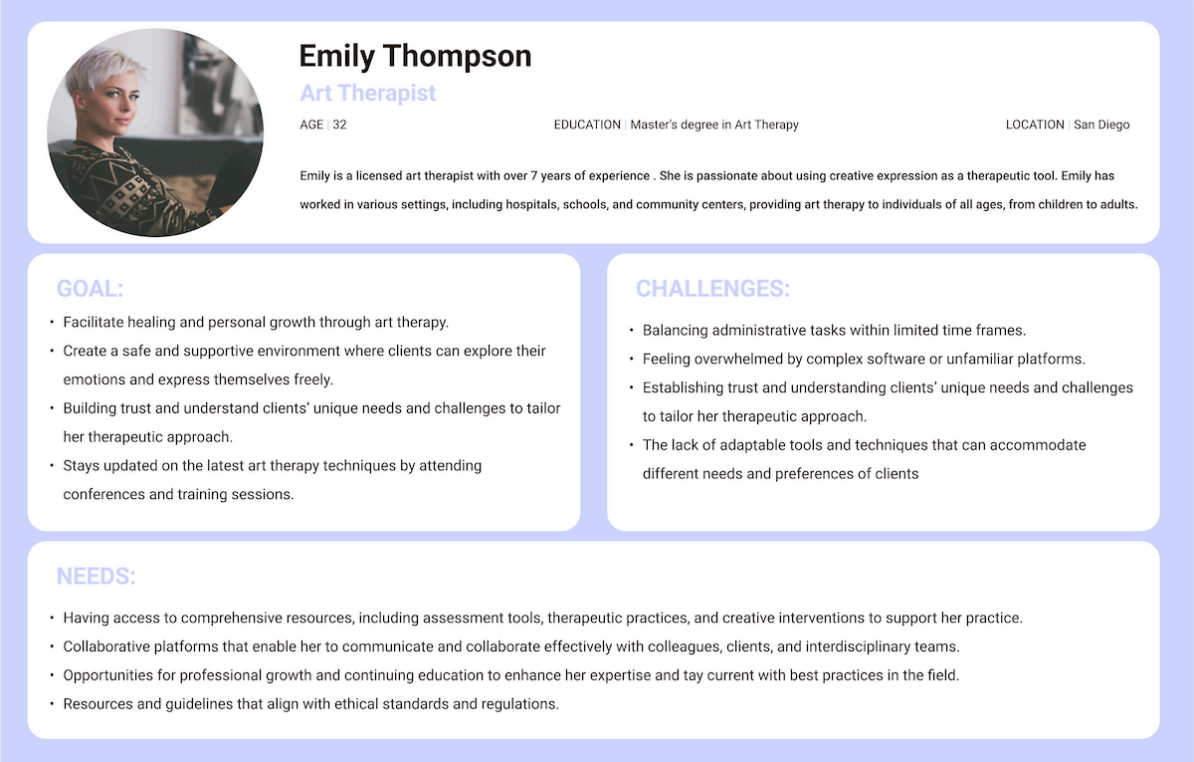
Emily’s persona. This image illustrates the persona used in the development of co-design sessions to ensure an effective design.

To gain a better understanding of our co-design workshop participants, we created a persona representing an art therapist. Personas are fictional characters that represent common user behaviors, helping designers gain insights into their target audience and ensuring a shared understanding within the design team. One significant advantage of having personas is their ability to keep designers focused on the core objectives of their target users, which enables them to prioritize both user and product requirements effectively [23]. Emily, a 35-year-old art therapist, is interested in using innovative approaches in her art therapy sessions. Developing her persona involved considering her goals, challenges, and needs in her practice (Figure 1).

### Concept Prototyping

In our study, we used AI generators, namely ChatGPT by OpenAI and Adobe Express, to develop a prototype for evaluation by art therapists. The prototype consisted of 2 main components: a series of cards and an AI-generated picture.

**Figure 2.**
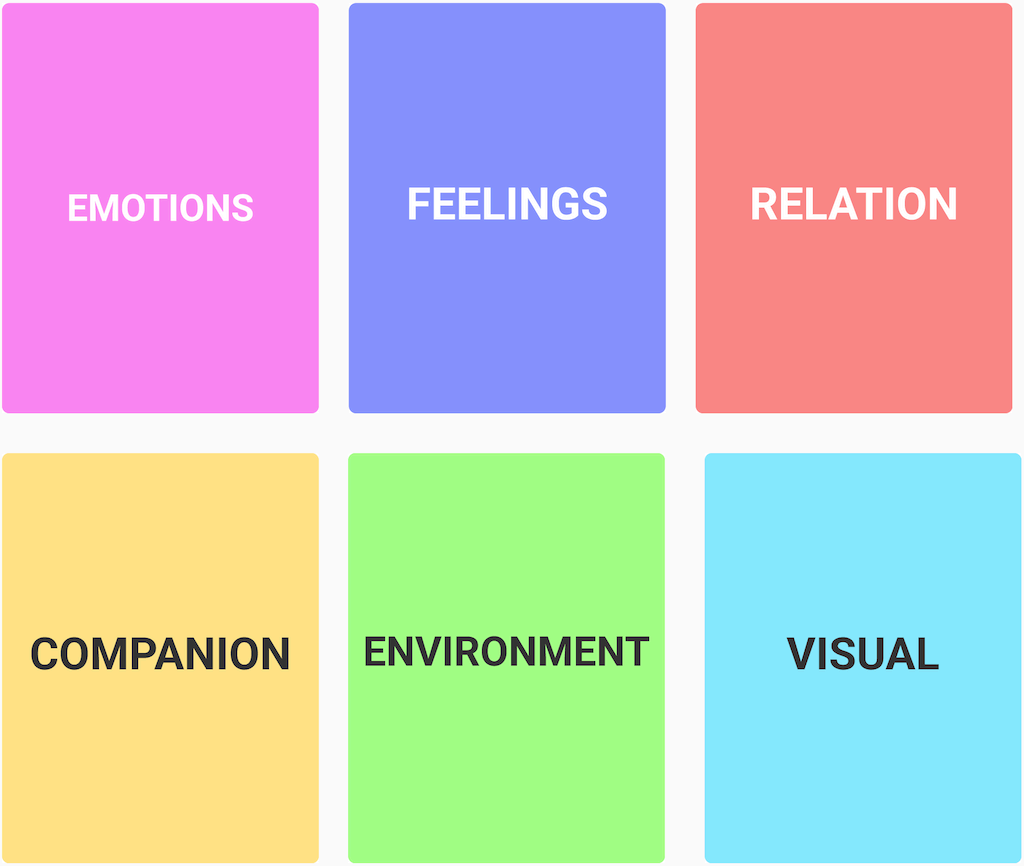
The categories of a list of words. This figure illustrates the 6 categories of emotions, feelings, relation, companion, environment, and visual for the list of words.

To begin the design process, we created a list of relevant words reflecting various emotions, drawing inspiration from Plutchik wheel of emotion [24]. Further, we expanded this list to include words of positive, negative, mixed or complex emotions, emotions of varying intensities, and those related to personal growth. Additionally, we incorporated words describing individuals or groups connected to the client, locations, and visual descriptors for environments aiming to cover all aspects of image creation. Finally, we categorize the words into 6 primary categories: emotions, feelings, relation, companion, environment, and visual. Each main category was further subdivided into subcategories to facilitate easy navigation and selection for both clients and therapists (Figure 2; see also Multimedia Appendix 3 for all categories and subcategories).

**Figure 3.**
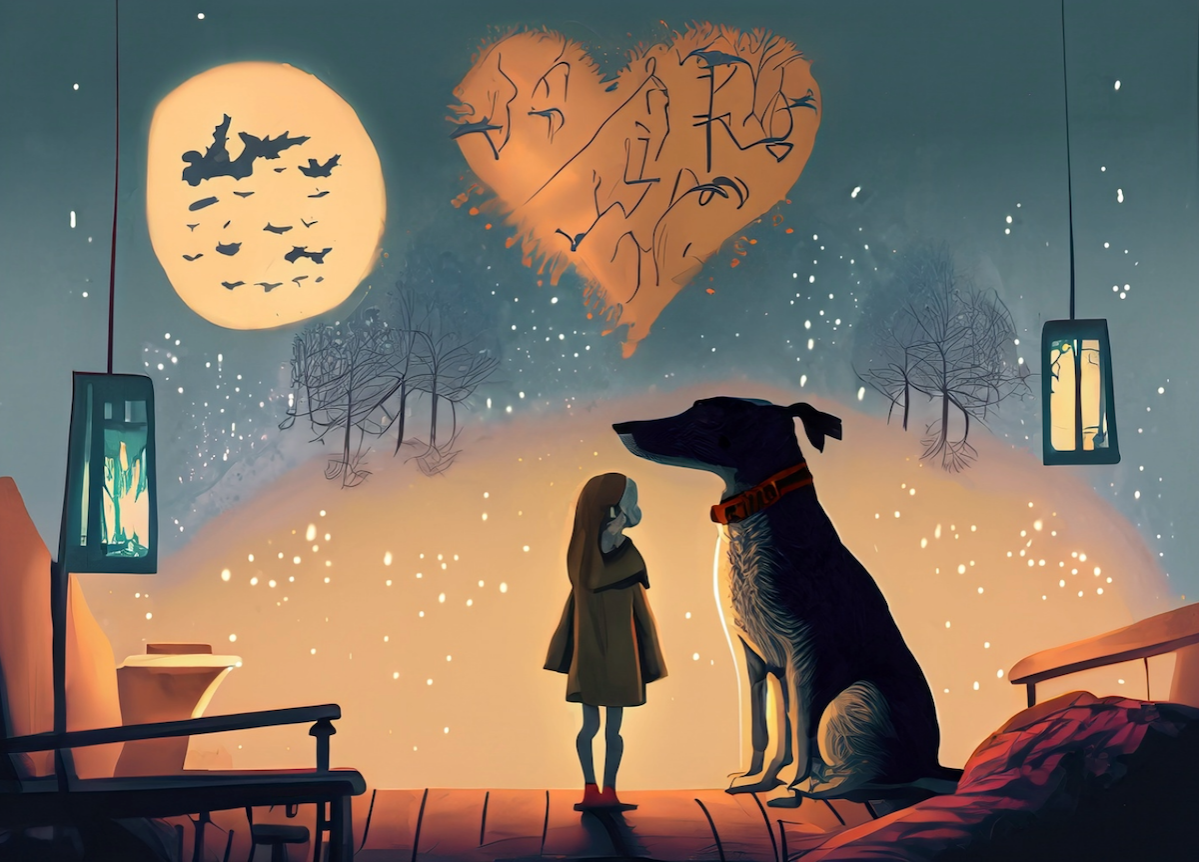
The AI-generated picture. This image was generated by the Adobe Express Generative AI to Text function, with “Demonstrate these feelings: love, hope, loneliness, conflicted, nostalgic, significant other, pet, home, night, fall, calm” as the prompt.

Subsequently, we used Adobe Express’s Generative AI Text to Image function to create a picture illustrating the selected emotions and feelings. We provided Adobe Express with a prompt listing specific emotions and contexts: “Demonstrate these feelings: love, hope, loneliness, conflicted, nostalgic, significant other, pet, home, night, fall, calm.” In response, we received 20 different images to choose from. For the final selection, we aimed to ensure that the chosen image effectively captured the specified emotions and contexts. By carefully selecting an image that best represented these feelings, we integrated the chosen image (Figure 3) with the cards, creating our final prototype. This prototype, including a deck of cards and an AI-generated image, was then presented to art therapists for feedback and evaluation. At the beginning of the co-design session, we presented the deck of cards and sought their insights on the effectiveness of the cards and their coverage of essential words. Afterward, we showed them the image, explaining that it was generated by AI using a random selection of words from the cards. We wanted to know whether they considered the image represented those words and if they thought they could determine the necessary information about the client from the image if it was a client’s work with AI.

### Recruitment

Participants were selected using a random sampling strategy at the American Art Therapy Association’s 54th Annual Conference. Informed consent was obtained verbally from all participants prior to their involvement in the study. Initially, 10 art therapists were recruited; however, data from one interview were excluded because the participant was not actively practicing art therapy at the time, resulting in a total of 9 interviews. The art therapists participated in one-on-one, in-person interviews, each lasting approximately 30 minutes. These interviews were scheduled at mutually convenient times and locations while maintaining participant confidentiality and voluntary participation.

Recruiting participants from this professional community through convenience sampling ensured their eligibility, providing time and cost savings compared to alternative approaches while offering diverse perspectives.

Based on insights from existing literature, the open-ended interview questions focused on three main themes: (1) the advantages of AI implementation in art therapy, (2) the disadvantages of AI implementation in art therapy, and (3) concerns regarding the use of AI in art therapy.

To ensure an accurate capture of participants’ responses, interviews were audio-recorded. These recordings were transcribed verbatim and subsequently anonymized by assigning participant code names (Participant 1, Participant 2, etc) to maintain confidentiality. The authors independently open-coded the transcripts and then collaboratively consolidated and established general codes. Following this, they individually identified child-codes and underwent a joint review and refinement process to ensure comprehensive coverage.

### Ethical Considerations

This study received approval from the institutional review board (IRB##21088) at Indiana University as exempt prior to participant recruitment. Verbal informed consent was obtained from all participants by FS before their participation, with the assurance that they could withdraw at any time without repercussions. Strict privacy and confidentiality protocols were adhered to throughout the study. All collected data were deidentified prior to analysis, ensuring participants’ identities remained confidential. Measures such as secure data storage and anonymization were implemented to protect participants’ privacy. Additionally, no images identifying participants were included in this manuscript, and all quotes used were preapproved with consent. Participation in the study was entirely voluntary, with no financial compensation or incentives provided to participants at any stage.

### Data Analysis

Data analysis was conducted using Braun and Clarke’s framework [25] to identify patterns in the coded data. We used Otter.ai for AI-driven interview transcription and Taguette, an open-source web-based document tagging tool, for code creation. FS approached the data without preconceived notions, coding the interviews to ensure an unbiased analysis. The coded categories were then cross-verified by FS, fostering an iterative process to identify relationships between codes and develop preliminary categories.

### Participants

Table 1 summarizes key demographic information about art therapists involved in the study. All participants had master’s degrees, while 22% (2/9) were registered art therapists, 33% (3/9) were board-certified registered art therapists, 11% (1/9) were licensed creative arts therapists, 11% (1/9) were registered provisional art therapists and licensed professional clinical counselors, 11% (1/9) were registered art therapists and licensed clinical professional counselors, and 22% (2/9) were waiting for their registered art therapist credentials.

**Table 1.** Participants’ demographics from semistructured interviews highlight their educational level, credentials, client age group, and client population.

Participant^a^	Education	Credentials	Client age group	Client population
1	Master’s	ATR^b^	School-age, teenager, and adult	Mental health
3	Master’s	ATR-BC^c^	All	Clinical
4	Master’s	ATR-BC	Children and adolescent	Behavioral health and acute hospitalized inpatients
5	Master’s	Pending ATR	All	General anxiety, depression, trauma, wellness, homelessness, and prison experience
6	Master’s	ATRP^d^ and LPCC^e^	12 years and over	All
7	Master’s	LCAT^f^	20 years and over	Forensic and transgender persons
8	Master’s	Pending ATR	All	Older adults
9	Master’s	ATR-BC	All	Development, behavioral, and quality of life needs
10	Master’s	ATR and LCPC^g^	Kids and young adults	Mental health, autism, neurodiversity, anxiety, depression, and grief

a Data from the interview with Participant 2 were excluded because the participant did not meet the study requirements and was not actively practicing art therapy at the time.

b ATR: registered art therapist.

c ATR-BC: registered art therapist–board certified.

d ATRP: art therapist registered provisional.

e LPCC: licensed professional clinical counselor.

f LCAT: licensed creative arts therapist.

g LCPC: licensed clinical professional counselor.

## Results

### Principal Findings

The analysis of the interview data revealed two main areas central to our study: (1) the integration of AI in art therapy and (2) using cards and AI-generating tools in art therapy practices. By exploring these areas, we gained insights into art therapists’ perspectives on AI’s role in art therapy, understanding potential benefits and considerations, categorized into three key themes: (1) is AI a substitute for traditional art therapy, (2) fostering a client-centered approach, and (3) enhancing health care access.

### Is AI a Substitute for Traditional Art Therapy?

Participants highlighted that AI could serve as a valuable medium to overcome resistance or concerns toward traditional art therapy practices. Participant 1 stated, “...[AI] would be helpful in jumping that threshold that people that hold back and they don’t want to make art...who kind of stay with the ‘I’ve never made art, I’m not an artist’...So that could potentially bridge the gap!”

Our findings suggested that AI empowers clients by creating a supportive environment where they can freely explore their thoughts without judgment or fear. Participants believed that AI also offers practical assistance in decision-making processes related to thematic elements and material selection. Moreover, participants noted that AI can facilitate the creation of new symbolic representations, enriching the creative process. Furthermore, art therapists highlighted the use of cards specifically, coupled with AI generation tools, as a structured method to help clients navigate imposter syndrome and self-doubt. They believed that by offering a structured framework, these cards allow clients to explore and express their emotions and thoughts in a guided way, helping them gain confidence and a sense of agency in their emotional journey. As Participant 5 noted, “…it could definitely be helpful for people who have trouble making decisions on how they want to express themselves.…”

The integration of AI into traditional art therapy was seen as especially beneficial for tech-savvy clients seeking venues for creative expression. In our study, AI was also highlighted as a “facilitator” for initiating conversation and establishing connections between art therapists and clients, with AI-generated images sparking therapeutic dialogue. As Participant 8 commented, “...the image itself can bring in emotions and thoughts, and help a person be able to verbalize certain things.” The co-design sessions also revealed that cards promote alternative modes of communication. Their playfulness helps establish connections, encourage conversation, and create a shared language for emotional exploration. While the cards offer a broad array of words, therapists appreciated their specificity, which enhances their effectiveness in facilitating deeper connections with clients. Participant 10 mentioned, “I think it could be a good way in especially for those who are into the technology, that it might be a way for them to tell a story without kind of having to....”

Despite AI’s potential, participants in our study noted the challenges in adopting AI tools, particularly for therapists or clients less familiar with digital tools. As Participant 8 mentioned, “...with generational gap areas, [we] might not know how to generate AI or might not understand some of it.” In navigating this transition, therapists emphasized the need to maintain a familiar therapeutic environment to ensure clients feel secure and heard while using AI. There was also concern about keeping clients’ autonomy. Art therapists expressed caution that AI could unintentionally diminish the client-therapist relationship by minimizing the client’s creative contribution.

Participants also expressed doubts about whether AI-generated images could fully capture clients’ emotions and thoughts in the same way traditional art does, where clients independently select symbols and shapes. Additionally, cultural sensitivity emerged as another concern, as art therapists questioned whether AI-generated applications could appropriately reflect the diverse cultural backgrounds and expectations of their clients.

*I think there are still a lot of cultural things to consider in using AI, especially if there are dialectical differences that people may have, or perceptual differences.*

[Participant 9]

The incorporation of AI in therapeutic contexts has raised ethical concerns among art therapists, particularly with regard to ownership rights, privacy, and confidentiality. Art therapists stressed the importance of protecting client data while reducing the possibility of unauthorized access. Discussions also focused on ownership rights related to AI-generated works and questions of authenticity within this new paradigm.

### Fostering a Client-Centered Approach

The use of AI technology in therapy settings was seen by art therapists as a key to fostering client empowerment and autonomy. Art therapists recognized the importance of allowing clients to interact independently with AI-generated tools, which not only opened up diverse creative possibilities but also encouraged a more personalized, self-directed therapeutic experience. As one of the participants highlighted, “client’s choice matters, and [art therapists] always give a choice to clients” [Participant 6]. This client-centered approach was regarded as fundamental to enabling clients to take ownership of their therapeutic journey, providing a sense of agency over the creative process.

Moreover, art therapists emphasized the empowering role of integrating cards into therapy sessions. By enabling clients to select and combine words that prompt AI-generated imagery, therapists believed this process could foster autonomy and boost client confidence in their expressive capabilities. Data from the study revealed that this iterative process not only broadens the scope of creative possibilities but also fosters a sense of ownership over the therapeutic journey. This combination of choice and structure, from art therapists’ point of view, helps clients feel more in control of their emotional exploration.

At the same time, participants expressed concerns about the accessibility and comprehensibility of cards, particularly for younger clients or those with cognitive impairments. For example, younger clients may struggle to interpret symbolic meanings due to their cognitive development stage, and individuals with cognitive impairments may find it difficult to process abstract concepts. As one art therapist noted, “...it was just amazing to me, but how does that come about for people who may not have the verbiage or don’t have the language?” [Participant 7]. The absence of linguistic cues in the cards poses an obstacle, making it challenging for certain clients to engage fully with the therapy process.

In addition, participants offered recommendations for optimizing the design and use of cards combined with AI tools. Art therapists stressed the importance of incorporating client-centered AI tools in therapy sessions, ensuring that clients feel like active participants in their treatment. As one art therapist put it, “...Through the collaborative process with the client, always getting their input, making them feel like a partner in the process, as opposed to being the one lead, they can almost act as the guide” [Participant 1]. Art therapists also suggested allowing clients to customize their cards with additional words and modify generated images would further empower them in their therapeutic process. Additionally, therapists recommended incorporating a variety of artistic styles into the cards to suit individual preferences and improve the effectiveness of therapy.

Participants also recommended a balanced, mixed approach to incorporating AI tools in therapy sessions, suggesting their use as supplementary aids rather than primary components. They proposed initiating therapy sessions with AI tools to engage clients therapeutically, followed by traditional art-making materials for deeper exploration. They believed that this approach would provide clients with a comprehensive and fulfilling therapeutic experience that combines the benefits of both technological innovation and traditional artistic expression.

Art therapists also emphasized the value of integrating self-evaluation tools alongside AI technology. They recommended incorporating Likert scales and pre- and postquestionnaires within art therapy sessions following the use of AI tools, allowing clients to reflect on their artwork and their monitor progress. One art therapist suggested, “...if you started here, and weeks later, making another image...maybe the computer can label they started with these feelings and words. You can rate as they were on the negative scale. And then here they are in the positive” [Participant 4]. Furthermore, art therapists proposed integrating a feature within AI tools that enables clients to compare their current artworks with those from previous sessions, fostering greater self-awareness and helping track personal growth.

Lastly, simplicity in design emerged as a key consideration for enhancing the usability of card-based AI tools. Art therapists recommended organizing words using the wheel of emotions to make the selection easier for clients and proposed categorizing words into positive and negative themes to guide emotional exploration. Preferences varied regarding the presentation of cards, with some art therapists advocating tactile physical cards for a more hands-on experience, while others preferred digital lists on screens for ease of selection and visualization.

### Enhancing Health Care Access

Participants in our study highlighted the potential of AI-generated platforms in improving the accessibility and effectiveness of virtual art therapy sessions. Through AI technology, therapists can seamlessly conduct sessions remotely, minimizing logistical obstacles associated with traditional art supplies. Additionally, the portability and accessibility of AI tools, accessible via electronic devices, increase their use across diverse therapeutic settings. Moreover, from the perspective of art therapists, these AI-driven tools offer valuable advantages to clients with physical impairments, helping them overcome the difficulties associated with handling conventional art supplies. As one participant mentioned, they would use it “if [the] client might have a disability or some challenges with identifying feelings” [Participant 9].

Particularly, participants highlighted the portability and convenience of incorporating cards with AI-generating tools into therapeutic sessions, emphasizing their capacity to overcome logistical barriers associated with traditional art supplies. With the ability to participate in therapeutic activities without the burden of physical materials, this accessibility is especially beneficial for clients with cognitive or physical impairments. Moreover, the structured format of the cards simplifies the process of selecting words, offering a user-friendly alternative for individuals facing cognitive impairments. One participant stated the value of this design, noting, “They [clients] may not have the emotional vocabulary. So, this is a helpful guide to give them something to bounce off of. Also, for older adults, with different forms of dementia, this could be really useful as a prompt for people who might have memory issues” [Participant 1].

## Discussion

### Principal Results

The findings from our study revealed that the integration of AI into art therapy holds significant potential to enhance both the accessibility and personalization of therapeutic practices. We discovered that AI could serve as a supportive tool, complementing rather than replacing traditional art therapy methods. Additionally, our study highlighted the promotion of a client-centered approach, where AI tools empower clients to explore their creative processes with greater autonomy. Insights from our co-design sessions further emphasized AI’s role in enhancing health care access, particularly for clients with physical or cognitive impairments, by reducing logistical barriers and providing more inclusive therapeutic options. From the analysis of our co-design sessions and semistructured interviews, we identified three primary areas for further improvement: (1) maintaining a balanced integration of AI in art therapy, (2) placing clients at the heart of art therapy practices, and (3) innovation for facilitating access to art therapy.

### Maintaining a Balanced Integration of AI in Art Therapy

Our study highlights the potential of integrating AI into art therapy to improve the therapeutic process. We identified that traditional art therapy sometimes falls short for clients who cannot express themselves artistically or verbally, which aligns with previous research findings [2]. Previous studies have also noted that art therapy is especially applicable for using technology, mainly because it facilitates easier sharing of images through digital platforms and reduces the dependence on verbal communication, while the use of symbols, metaphors, and projections in art therapy remains effective regardless of the medium used [9,26,27]. In addition, our findings build upon this by demonstrating that AI can act as a bridge for these clients, offering novel avenues for self-expression, particularly assisting those who may feel inhibited due to a perceived lack of artistic skill or fear of judgment which is a common barrier in traditional art therapy sessions [2].

Another challenge in traditional approaches in art therapy according to our participants is the struggle of starting the conversation. Our co-design sessions with art therapists revealed that AI can enhance communication and foster emotional expression, especially for clients who struggle to verbalize their emotions. Art therapists emphasized the importance of relationship-building and initiating conversations, particularly with clients who are resistant to talking. Using digital media in art therapy sessions can facilitate adaptation, communication, and relationship formation, offering diverse expression methods and promoting participant satisfaction and achievement [4]. The results from our co-design sessions show that our design helps clients initiate conversations in a playful manner using cards, allowing them to explore multiple categories and generate images based on their choices. Studies also show that AI tools provide an essential avenue for expression beyond words, benefiting those with limited verbal communication skills [2,3]. While indicating that generative algorithms help clients materialize their imaginations and emotions, they also facilitate communication through generated images [28,29]. Therefore, our design combining cards with AI-driven tools can address these barriers.

However, our findings show that the transition to digital tools is not without challenges. Therapists raised concerns about the sensory limitations of digital artwork, which can detract from the therapeutic experience, as noted in previous studies [30-32]. Therefore, we believe that depending solely on technology is not a correct approach in art therapy. Our design with tangible cards of words in addition to AI showed that it can tackle this struggle. Instead of using art supplies, clients can come up with an AI-generated image based on choosing cards. They can also change the prompt or add to it to change the image, the colors, and even the lighting, like what they can do with art supplies in the traditional approach but easier. We were trying to maintain the balance between traditional and technology by this design concept. It is essential to ensure that both tech-savvy and non–tech-savvy clients benefit from these innovations. In our study, we also found that ethical considerations are another concern, including issues of privacy, authenticity, and therapist competency. In our design, we have not addressed this concern fully, and our current concept focuses on image generation without covering data storage. However, for future iterations, we believe it is essential to adhere to ethical frameworks of using AI in health care with guidance from health care professionals to ensure client safety while respecting autonomy, data security, confidentiality [16], and maintaining the authenticity of AI-generated images, all of which are critical challenges in integrating AI into art therapy sessions. As Zubala et al [9] noted, while the exploration is vital for maintaining the relevance of art therapy, it is also important to recognize that “art therapy is eclectic and not reducible to a single set of algorithms.”

### Placing Clients at the Heart of Art Therapy Practices

Art therapy revolves around tailoring sessions to meet individual client needs and preferences. Therefore, it is crucial that technological interventions, such as AI, support rather than diminish client autonomy and decision-making [33]. In our design, by incorporating cards and initiating discussions based on AI-generated images, aiming to place clients at the center of the therapy session, they are able to choose words reflecting their thoughts and emotions and a tangible starting point to explore their feelings and experiences. Our findings indicate that this approach was successful, as art therapists believed it provided clients with confidence and autonomy, enabling them to guide the AI prompts according to their cards’ preferences. Moreover, our design does not need a high digital literacy to be used by art therapists or clients, which is an important challenge in introducing digital interventions in art therapy [2]. Tools such as AI decision-support systems and AI-assisted web-based therapy platforms, as proposed by previous research [5,34-36], aim to enhance therapist-client interactions and empower clients by actively involving them in the session process. Using cards as a session starter shows a playful connection between art therapists and clients, which art therapists believed that it is particularly beneficial for clients who may struggle to articulate their emotions or discuss their condition in the beginning. However, concerns raised by art therapists in our study suggest that the use of AI may inadvertently diminish client ownership and control over the therapeutic process. To address this, our findings suggest providing clients with options to customize prompts, allowing them to add words if they cannot find suitable ones among the provided cards to increase their autonomy; giving clients the autonomy to modify generated AI images can help mitigate these concerns.

In addition, aligning with the user-centered design approach [37,38] and placing the client at the center of the art therapy process, we realized the importance of self-evaluation, as our initial design lacked giving the clients feedback on their progress. Therefore adding a component to allow clients to perform self-evaluation after each session and compare their artwork to previous sessions is needed for further designs. Additionally, while integrating AI in art therapy sessions helps clients gain more control and direct the sessions, from our study, we discovered that we must avoid adding complexity through technology. Art therapists also mentioned that our design needs to increase simplicity by adhering to a user-centered design framework [39,40] to make the cards usable and understandable for clients across all literacy levels and clients with lower cognitive skills. Each introduced technology should reduce the burden on clients and art therapists, serving as an assistive tool [2].

### Innovation for Facilitating Access to Art Therapy

Studies show that integrating technology into art therapy sessions offers a feasible alternative to traditional physical art materials, expanding therapeutic opportunities and improving access to art therapy [2,7,9,41,42]. Aligning with previous research, our design demonstrated AI’s potential to remove barriers and enhance accessibility in art therapy sessions. This barrier could be because of the transportation to the art therapy session, carrying art supplies, or financial problems of clients with regard to buying art materials. Sometimes, having art supplies present in art therapy sessions is unsafe for some groups of clients [2]. Solving these issues, art therapists in our study highlighted AI’s potential to facilitate sessions, especially telehealth, noting the ease of using digital materials compared to transporting physical art supplies. The portability afforded by technology has long been studied and holds promise for improving accessibility to therapy [2,32,43,44].

Our design incorporating AI offers another alternative for clients with physical and cognitive impairments, empowering them to engage in the art-making process. Our study showed the benefit of using cards, including organized word categorization, particularly for clients with cognitive impairments, to facilitate expression and communication. Similarly, AI-generated tools proved beneficial for clients with physical impairments who face challenges in using traditional art supplies, a finding supported by prior research [2].

Despite the benefits, we believe that challenges remain in the adoption of AI in art therapy. It is crucial to recognize that clients and art therapists may lack proficiency in using technology [2,4]. Therefore, it is essential to ensure that AI serves as an assistive tool for both art therapists and clients, alleviating rather than exacerbating difficulties in the therapeutic process, particularly for clients with physical or cognitive impairments. In addition, lacking internet access or access to technology can be another concern for using our concept. While using technology might be challenging for some clients, due to a lack of digital literacy, using AI can be challenging for increasing access to health care.

### Limitations

We believe that while our study provides insights into the integration of AI in art therapy, several limitations necessitate further research to fully explore its potential and address key concerns. We propose the following recommendations for future studies. Our study exclusively focused on the experiences of art therapists. Future research should actively involve clients to gather their perspectives on the use of AI and our design in art therapy sessions. The methodology used in our study limited our ability to explore the practical application of our design in real art therapy sessions. Subsequent research should prioritize testing the integration of our design in authentic therapeutic environments. This approach will enable researchers to examine the dynamics of interactions between art therapists and clients using AI-assisted tools, providing insights into its efficacy and potential challenges. Our design requires iterative refinement based on feedback from art therapists to enhance its usability and effectiveness. It is vital to simplify categories and optimize language to ensure accessibility for a diverse range of clients. In order to assess the effectiveness of our design within art therapy environments, assessment frameworks in addition to ethical frameworks should be implemented. These frameworks should include self-evaluation methods for clients, as well as mechanisms for art therapists to securely track clients’ progress over time. Our research showed that incorporating AI-generated images and words from previous sessions can facilitate ongoing assessment and intervention refinement.

As a result, future studies investigating the use of AI in art therapy need to apply a multifaceted approach, taking into account the viewpoints of both clients and therapists. Future research can expand on this study of the potential of AI in therapeutic practice by addressing these limitations and applying our recommendations into practice.

### Conclusions

In this study, we explored the integration of AI into art therapy by conducting co-design sessions and semistructured interviews with art therapists. Our design featured a deck of cards with words in 6 categories covering emotion, feeling, relation, companion, environment, and visual, paired with an AI-generated image based on a prompt with selected words. We used AI tools such as ChatGPT (OpenAI) and Adobe Express to generate an image that was specifically designed for use in art therapy sessions. By merging AI’s capabilities with insights from art therapists and human-computer interaction design researchers, we aimed to improve the therapeutic experience for clients and therapists. Our findings suggested that AI can act as an assistive tool, particularly for clients who struggle to express themselves verbally or artistically. However, the use of AI in art therapy presents challenges, including ethical concerns regarding data security and client confidentiality. Future research is needed for more comprehensive testing in clinical settings including clients. By addressing these considerations, we believe that AI can play an important role in making art therapy more inclusive and accessible for diverse groups of clients.

## Supplementary material

10.2196/63038 Multimedia Appendix 1 Co-design protocol for artificial intelligence in art therapy study.

10.2196/63038 Multimedia Appendix 2 Semistructured interview questions.

10.2196/63038 Multimedia Appendix 3 Cards’ categories and subcategories.

## Abbreviations

AI: artificial intelligence
CAT: creative arts therapy
