# Supplementary material for: Insights From Art Therapists on Using AI-Generated Art in Art Therapy: Mixed Methods Study
Source: JMIR Form Res. 2024 Dec 4;8:e63038. doi: 10.2196/63038 (PMC11634044; doi:10.2196/63038)
Supplement: Multimedia Appendix 1 [file formative-v8-e63038-s001.doc]

## Co-Design Protocol for AI in Art Therapy Study

### Introduction:

Participants are invited to take part in a research study exploring the use of AI during art therapy sessions. The study aims to identify opportunities for improving technologies used in therapeutic settings. Participation is voluntary, and the session will last approximately 30-45 minutes, followed by a few interview questions.

### Study Process:

1. Introduction to the Study: At the start of the session, participants will be introduced to the study team’s member, the purpose of the study and how AI-generated images can potentially be used in art therapy to inspire and support therapeutic processes.

2. Presentation of Materials: Participants will be shown a series of cards organized into six categories, each containing words related to emotions, feelings, relation, companion, environment, and visual. They will also be shown an AI-generated image created by selecting words from these categories.

3. Explanation of the AI-Generated Image: The facilitator will explain that the image presented was generated by selecting specific words from the categories, and these words influenced the visual output produced by the AI system.

4. Feedback on the Image and Word Categories: Participants will be asked to reflect on the AI-generated image and share their thoughts on its relevance and potential use in therapy sessions.

5. Reordering and Adjusting Categories: Participants will be invited to re-order the words within the categories or suggest any words that could be added or removed. Their insights will help refine the categories and word selections for future AI image generation.

6. Interview and Discussion: After the co-design activity, a semi-structured interview will be conducted to gather further insights.

7. Conclusion: The session will conclude with a brief wrap-up, where participants can share any additional thoughts or ideas that arose during the co-design activity.

### Consent and Voluntary Participation:

Participation is voluntary, and participants may withdraw at any time.

### Closing Line:

Thank participants for their valuable contributions to this study.
