# Supplementary material for: Insights From Art Therapists on Using AI-Generated Art in Art Therapy: Mixed Methods Study
Source: JMIR Form Res. 2024 Dec 4;8:e63038. doi: 10.2196/63038 (PMC11634044; doi:10.2196/63038)
Supplement: Multimedia Appendix 2 [file formative-v8-e63038-s002.doc]

## Semi-Structured Interview Questions

### 1. Education:

What is the highest level of education you have attained? (Please select and fill in the blank)

- High School _______________________________________________
- Bachelors in _______________________________________________
- Masters in ________________________________________________
- Doctorate in _______________________________________________
- Other _____________________________________________________

### 2. Current Art Therapy Credential:

- ATR-BC
- ATR
- Completing Registration Hours
- No Art Therapy Registration

### 3. Age Groups Served:

Which age groups do you work with? _______________________________________________________________________________

### 4. Populations Served:

Which populations do you serve? (Dementia, grief, mental health, etc.) _______________________________________________________________________________

### 5. AI-Generated Image Use in Therapy:

We have generated this picture using generative AI. We used cards from these 6 categories [hand them the cards], chose specific words, and provided them to the AI tool. Here is the result [hand them the picture]. How do you think it could be helpful if this image were one of your clients' works during an art therapy session? _______________________________________________________________________________

### 6. Evaluation of Card Categories:

Please review the cards. Do they make sense for use in an art therapy session? _______________________________________________________________________________

### 7. Additional Categories or Sub-Categories:

Is there any other category or sub-category you think we should add to these? _______________________________________________________________________________

#### A. Therapeutic Process Enhancement:

What aspects of the therapeutic process can be enhanced or complemented by incorporating generative AI and digital art creation? _____________________________________________________________________________________ _____________________________________________________________________________________

#### B. Additional Categories/Sub-Categories:

What other categories and sub-categories could you think of adding to this collection? _____________________________________________________________________________________ _____________________________________________________________________________________

#### C. Aligning Keywords with Therapeutic Goals:

How can we ensure that the keywords chosen to represent emotions align with therapeutic goals and the unique needs of each client? _____________________________________________________________________________________ _____________________________________________________________________________________

#### D. Guidelines for Safe Therapeutic Environment:

What guidelines or constraints should be in place to maintain a safe and effective therapeutic environment while using generative AI for art creation? _____________________________________________________________________________________ _____________________________________________________________________________________

#### E. Feedback and Monitoring Mechanisms:

What feedback mechanisms and monitoring processes can be established to assess the impact and effectiveness of this approach on clients' emotional expression and well-being? _____________________________________________________________________________________ _____________________________________________________________________________________
