# Supplementary material for: Insights From Art Therapists on Using AI-Generated Art in Art Therapy: Mixed Methods Study
Source: JMIR Form Res. 2024 Dec 4;8:e63038. doi: 10.2196/63038 (PMC11634044; doi:10.2196/63038)
Supplement: Multimedia Appendix 3 [file formative-v8-e63038-s003.doc]

## Cards’ Categories and Sub-Categories

Using this list of words, we utilized Figma platform to categorize the words into six primary categories: Emotions, Feelings, Relation, Companion, Environment, and Visual. Each main category was further subdivided into sub-categories to facilitate easy navigation and selection for both clients and therapists.


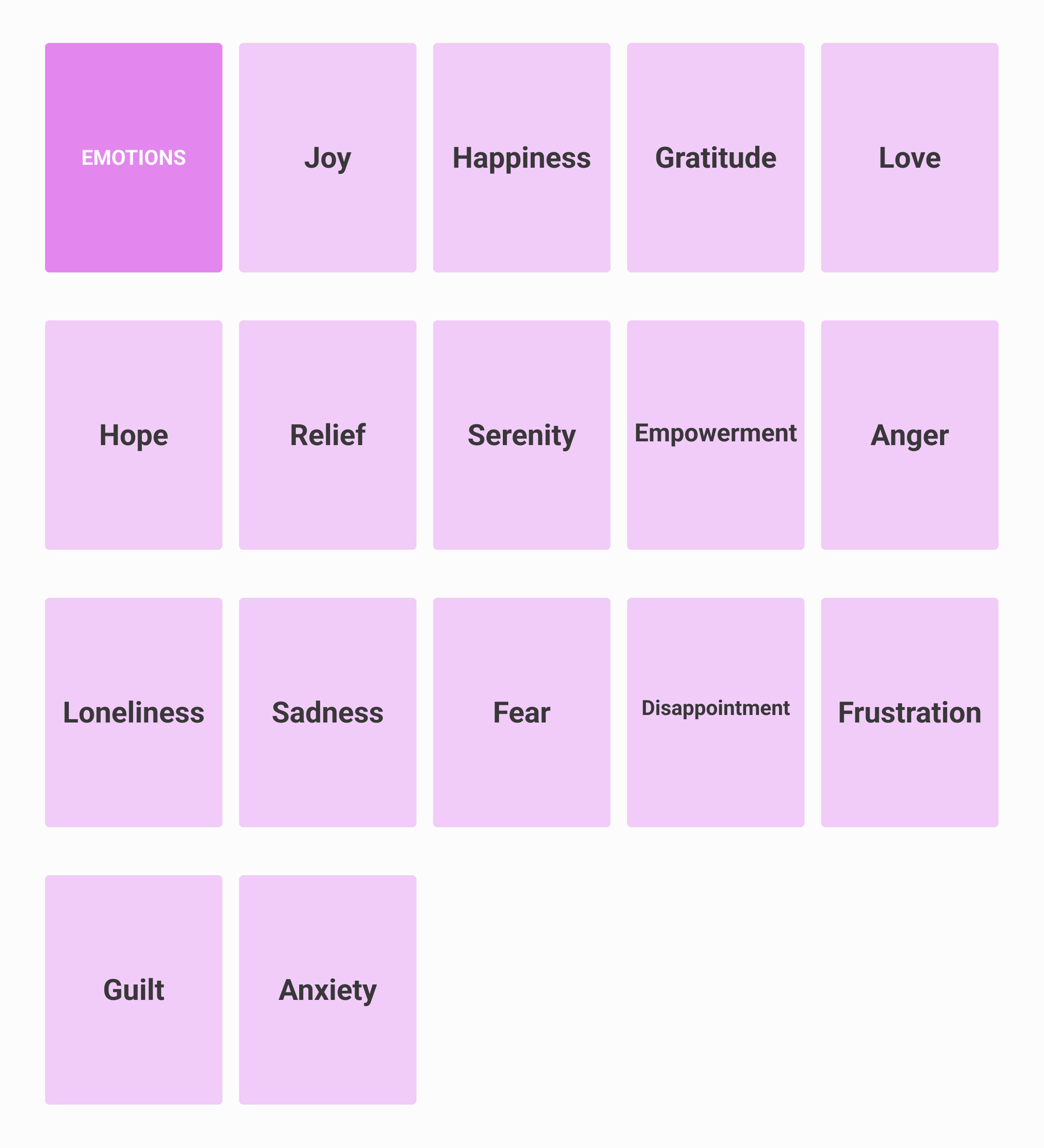


Figure 1. "Emotions” category and subcategories.


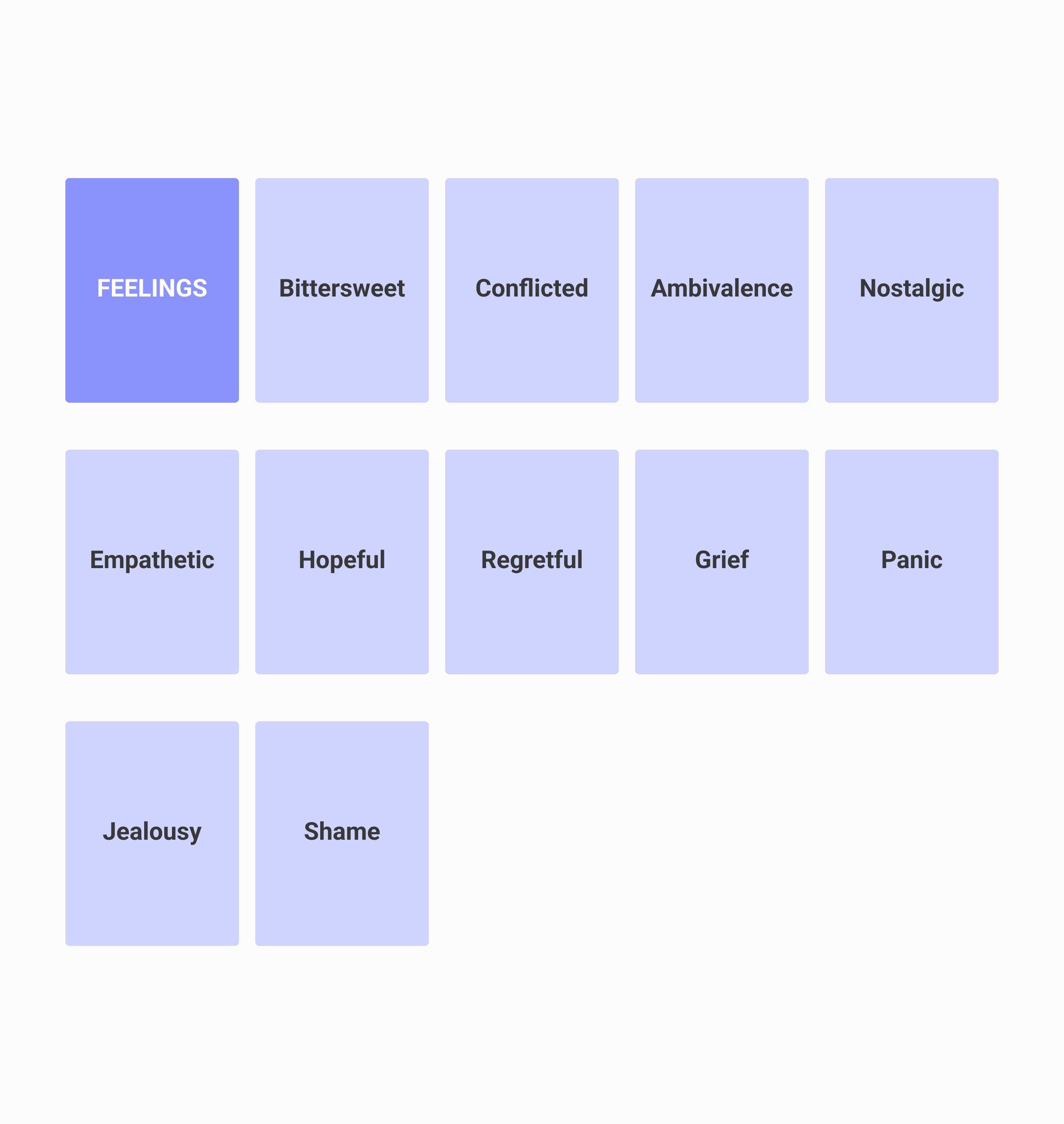


Figure 2. "Feelings” category and subcategories.


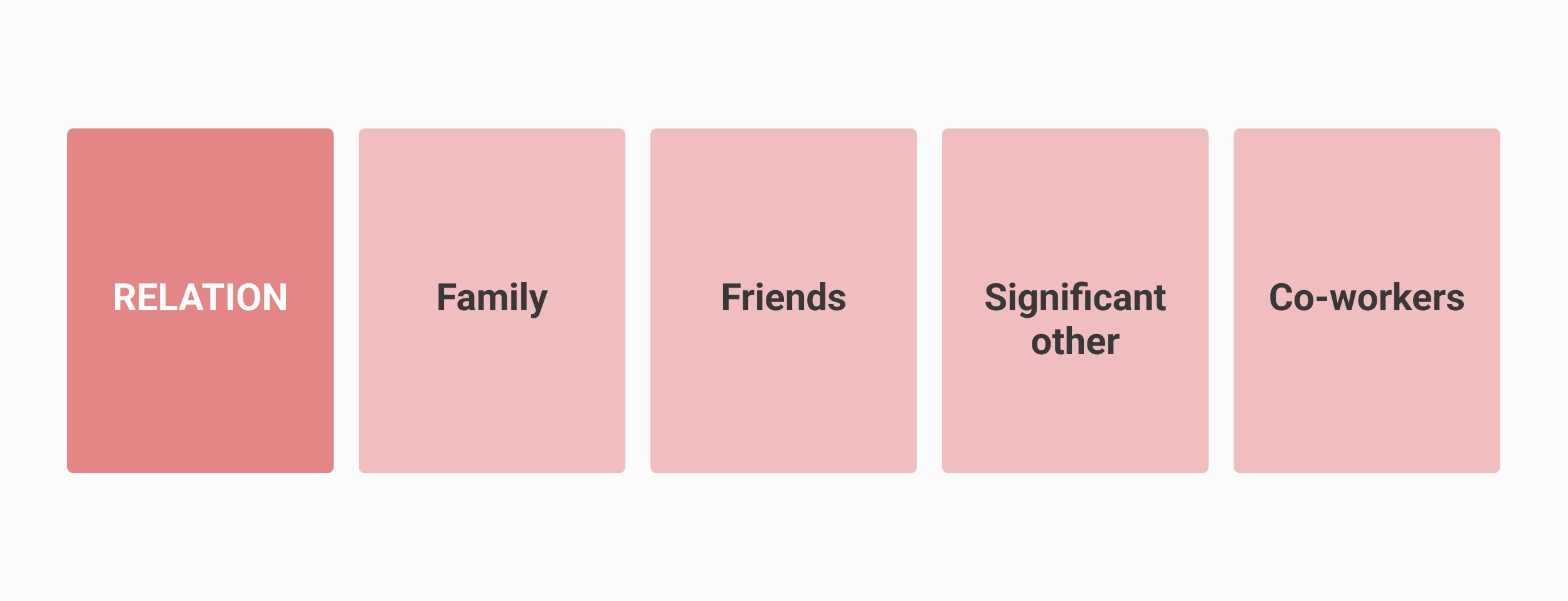
Figure 3. "Relation” category and subcategories.


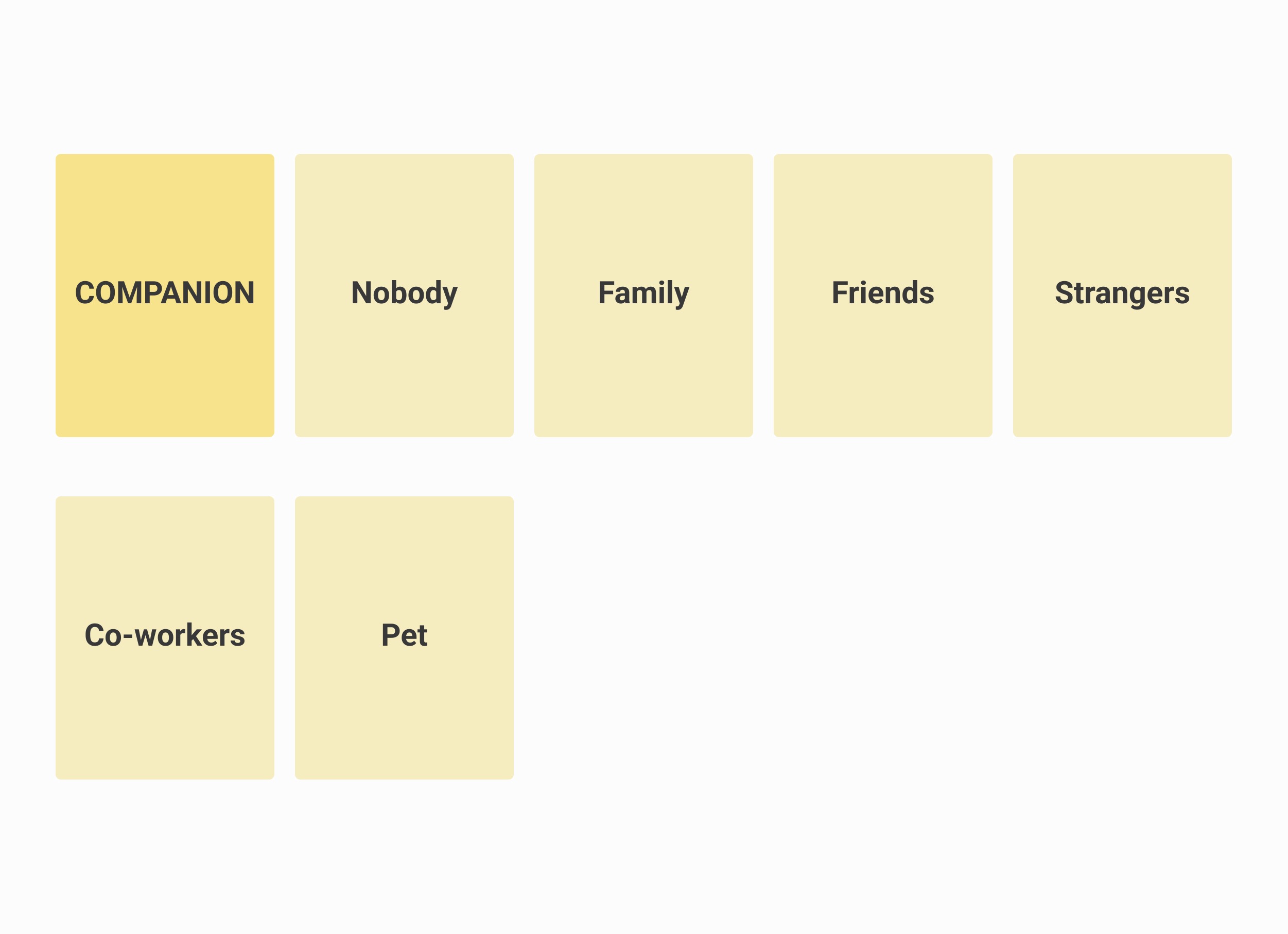
Figure 4. "Companion” category and subcategories.


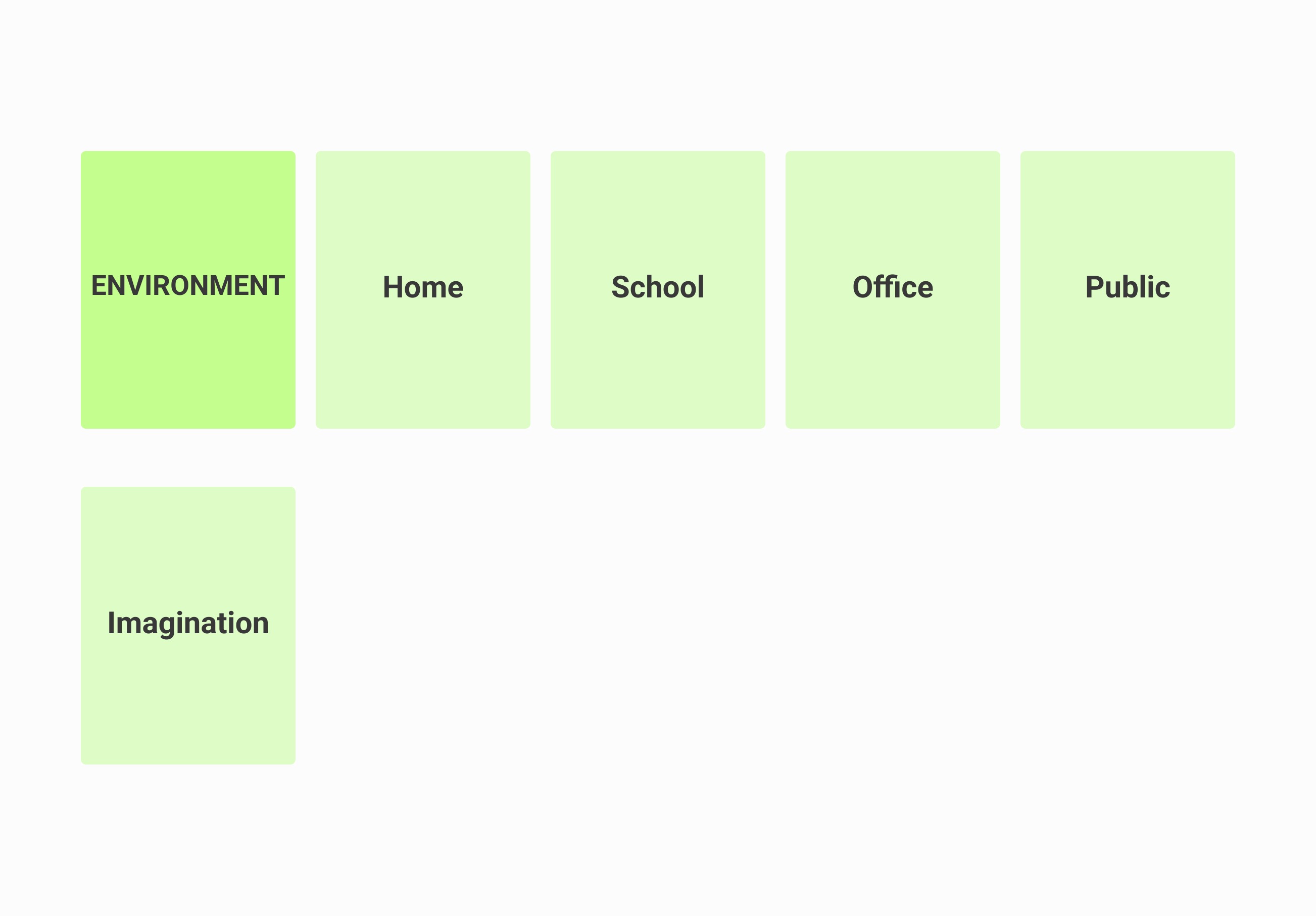
Figure 5. "Environment” category and subcategories.


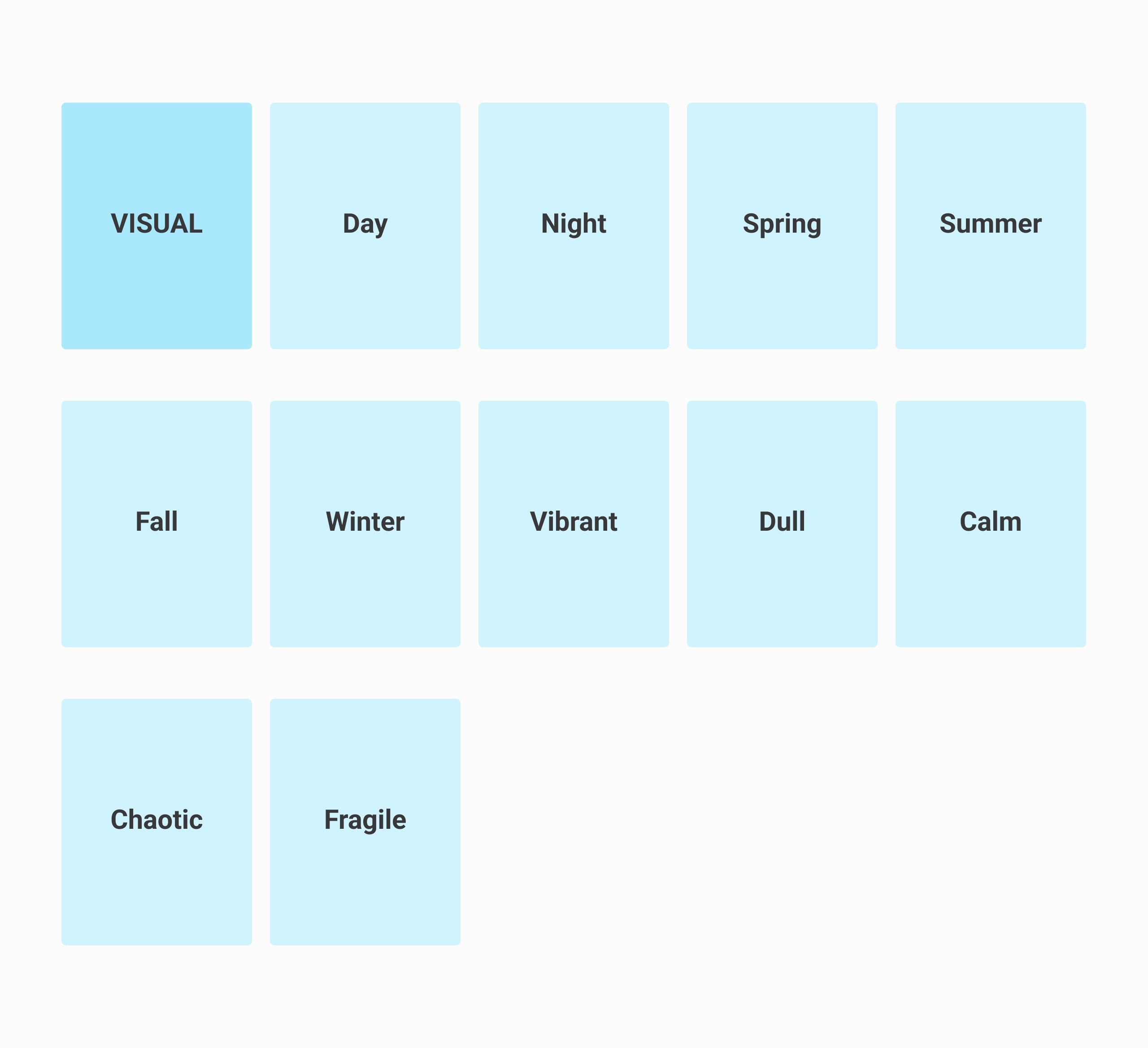
Figure 6. "Visual” category and subcategories.

As depicted in the above figures, each category is represented by cards containing words relevant to that specific category. Under the emotions category, sub-categories included "Joy, Happiness, Gratitude, Love, Hope, Relief, Serenity, Empowerment, Anger, Loneliness, Sadness, Fear, Disappointment, Frustration, Guilt, Anxiety." Feelings were categorized into sub-categories such as "Bittersweet, Conflicted, Ambivalence, Nostalgic, Empathetic, Hopeful, Regretful, Grief, Panic, Jealousy, Shame." Relations sub-categories encompassed "Family, Friends, Significant other, Co-workers," while companionship sub-categories featured "Nobody, Family, Friends, Stranger, Co-workers, Pet." Environmental categories comprised cards representing "Home, School, Office, Public, Imagination" and "Day, Night, Spring, Summer, Fall, Winter, Vibrant, Dull, Calm, Chaotic, Fragile."
